# Molecular Dynamics—From Macromolecule to Small Molecules

**DOI:** 10.3390/ijms23105676

**Published:** 2022-05-19

**Authors:** Ki Hyun Nam

**Affiliations:** 1 Department of Life Science, Pohang University of Science and Technology, Pohang 37673, Korea; structures@postech.ac.kr; 2 POSTECH Biotech Center, Pohang University of Science and Technology, Pohang 37673, Korea

All natural molecules have their own physical, chemical, or biological properties and functions. To accurately understand the properties and functions of these molecules, it is important to first observe their structure and then their molecular dynamics [1]. Molecular dynamics are observed in all molecules, from small molecules to macromolecules, and provide information about their intrinsic structure in a spatiotemporal context within a specific environment. Studies in molecular dynamics provide useful and important information in understanding the properties and functions of molecules and their spatial movement over time. The target molecules used in research have different physicochemical properties, and accordingly, the spatial and temporal information of the molecule to be observed is different, as well as the applied experimental technique. Through the observation and prediction of molecular dynamics, we can accurately understand the entire process of the chemical and physical mechanisms of these molecules. Understanding these processes contributes not only to academic knowledge, but also to potential applications in various fields such as the materials industry and medicine. To understand the unique properties of both small- and macro-molecules and chemical or biological samples in nature, specific scientific tools, and laboratory techniques have been applied [1]. Although the characteristics and experimental approaches of small molecules and macromolecules in the natural environment are different, this Special Issue has dealt with various research fields using spectroscopy, crystallography, liquidography, and computational studies under the general theme of molecular dynamics. In this Special Issue, we discuss molecular dynamics tools and related research results, covering small molecules to macromolecules. Specifically, we covered one editorial [1] and seven research articles [2,3,4,5,6,7,8], as follows below.

The importance of the structure and molecular dynamics of molecules has been explained in the opening editorial [1]. Additionally, this editorial covers a summary of the timescales of molecular motions and techniques for structure and dynamics [1]. This information will be helpful for the design of molecular dynamics research.

Serial crystallography (SX) using X-ray free-electron lasers or synchrotron X-rays is an emerging technique for determining the room-temperature structure of macro- and small-molecules [9]. This method allows us to visualize the time-resolved molecular dynamics of macromolecules using pump-probe experiments. Nam applied a lard material as a new sample delivery method for serial crystallography [2]. Using this method, a number of crystals were stably delivered into the X-ray interaction point, and the room-temperature structure of lysozyme and glucose isomerase was successfully determined. This method can be applied in serial crystallography studies to better understand the molecular fluctuations and dynamics of macromolecules at room temperature. 

Sarimov et al. studied the denaturation of lysozyme from hen egg whites after treatment with dithiothreitol and guanidine hydrochloride [3]. Using various modern optical tools such as interferometry, dynamic light scattering, and spectroscopy, they showed time-dependent structural changes occur not only in the structure of a protein, but also in its hydration shell. This provides important information in understanding the structural changes that occur over time in protein denaturation and folding.

Nam reported the room-temperature structure of glucose isomerase complexed with a xylitol inhibitor [4]. To provide more accurate structural information without radiation damage, serial synchrotron crystallography experiments were performed, and high-resolution crystal structures were determined. This study elucidated the mechanism by which xylitol inhibited the active site of glucose isomerase and how xylitol released metal ions at the active site. These results will be useful in understanding the molecular mechanism of glucose isomerase and will provide insights into its industrial application. 

Yang and Han explained the tunneling phenomenon that occurs in ammonia molecules from the perspective of trajectory-based quantum dynamics [5]. The vibration of the nitrogen atom in ammonia for the two equilibrium positions was analyzed using the Hamiltonian equations of motion. The vibration period was calculated using the quantum trajectory and was compared with the experimental measurements. This study analyzed the transition between the two states in detail, and proposed theoretical results for different tunneling ranges in the two states.

Gu et al. investigated the solvent effect on the photodissociation of C_2_F_4_I_2_ in cyclohexane via time-resolved X-ray liquidography (TRXL) [6]. This study revealed that fluorination affects the solvent dependence, reaction pathways, and molecular structures of the reaction intermediates. This study demonstrated that the TRXL technique is useful for studying solvent dependence in the solution phase.

Fan et al. reported the anti-Kasha behavior of 3-hydroxyflavone and its derivatives [7]. The photochemistry of 3-hydroxyflavone was studied using femtosecond transient absorption spectroscopy and interpreted using computational studies. With a higher energy excitation, the excited state intramolecular proton transfer (ESIPR) was not ultrafast, as was observed in lower energy excitations. 

Luis et al. reported the structural dynamics of soluble and membrane-bound interleukin-1 receptor type-1 (IL-1R1) with ectodomains (ECDs) using molecular dynamics (MD) simulations [8]. This study focused on the structural flexibility and spatial rearrangement of ECD. The simulations and analyses presented in this study provided insights into the structure and dynamics of IL-1R1, which may be explored in drug discovery.

Overall, this Special Issue covers a wide range of topics, from small molecules to macromolecules, including experimental techniques, research results, and computational analysis, which can contribute to the field of molecular dynamics as a whole. Finally, I would like to thank all the authors for providing excellent manuscripts, the reviewers, and editors for providing constructive feedback, and the editors of the *International Journal of Molecular Sciences* for working together on this Special Issue.
